# Malnutrition, Functional Decline, and Institutionalization in Older Adults after Hospital Discharge Following Community-Acquired Pneumonia

**DOI:** 10.3390/nu16010011

**Published:** 2023-12-20

**Authors:** Sandra Clotet-Vidal, M. Encarna Saez Prieto, Pol Duch Llorach, Álvaro Santos Gutiérrez, Jordi Casademont Pou, Olga H. Torres Bonafonte

**Affiliations:** 1 Internal Medicine Department, Hospital de la Santa Creu i Sant Pau, 08041 Barcelona, Spain; jordi.casademont@uab.cat; 2 Medicine Department, Universitat Autònoma de Barcelona, 08913 Barcelona, Spain; olga.torres@uab.cat; 3 Geriatrics Unit, Internal Medicine Department, Hospital de la Santa Creu i Sant Pau, 08041 Barcelona, Spain; esaez@santpau.cat (M.E.S.P.); asantosg@santpau.cat (Á.S.G.); 4 Infectious Diseases Unit, Internal Medicine Department, Hospital de la Santa Creu i Sant Pau, 08041 Barcelona, Spain; pduch@santpau.cat; 5 Biomedical Research Institute Sant Pau (IIB Sant Pau), 08041 Barcelona, Spain

**Keywords:** vitamins, nutrition assessment, micronutrients, malnutrition

## Abstract

Background and aims: Community-acquired pneumonia (CAP) is a major threat to older adults, but mid-term implications are poorly described. The aim was to analyze functional decline, institutionalization, malnutrition, and risk factors after hospital admission for CAP. Methods: This prospective observational study included patients over 65 years discharged after CAP between May 2019 and July 2021. We performed a comprehensive geriatric assessment and a general nutritional assessment 30–60 days after CAP. This included the MNA and blood test with trace elements and vitamins. The main outcomes were functional decline, institutionalization, and malnutrition. Multivariate logistic regression was used for the analyses. Results: In total, 144 patients of 77.15 ± 7.91 years, 55.6% male, and 9% previously institutionalized were analyzed. At hospital admission, the Charlson Comorbidity Index (CCI) was 1.5 ± 1.6, the Pneumonia Severity Index was 98.1 ± 25.9, and the previous Barthel Index (BI) was 93.06 ± 17.13. Hospital stay was 9.72 ± 7.88 days. After 44.6 ± 14.4 days, 48.6% patients showed functional decline and 19.4% were institutionalized. Age (OR 1.17; CI 95% 1.09–1.26), previous institutionalization (29.1; 3.7–224.7), BI (1.09; 1.05–1.14), CCI (1.5; 1.1–2.1), and length of stay (1.1, 1.02–1.18) were independently associated with functional decline. The only predictors of new institutionalization were previous BI (0.96; 0.93–0.99) and length of stay (1.06; 1.00–1.13). The MNA indicated malnutrition in 28% of the community-dwelling patients and 67.9% of those institutionalized, with risk of malnutrition being 45.7% and 9.5%, respectively, after an average of 44.6 days of CAP diagnosis. The predictors of malnutrition were previous institutionalization (10.62; 2.20–51.21), BI (0.95; 0.92–0.98), and length of stay (1.12; 1.04–1.20). Micronutrient deficiencies were mainly zinc (61.8%), vitamin D (54.5%), and vitamin C (45.1%). An MNA score < 17 points or hypoalbuminemia showed good specificity to identify these deficiencies. Conclusions: After CAP admission, functional decline, institutionalization, and malnutrition rates were high. Longer hospital stay was a common risk factor for all outcomes. The presence of hypoalbuminemia or an MNA < 17 in older patients should prompt suspicion of deficiencies in micronutrients, such as vitamin D, C, and zinc.

## 1. Introduction

Community-acquired pneumonia (CAP) remains an important cause of morbidity and mortality [1]. Furthermore, CAP is a major threat to older adults, probably because the age-associated decline in immune function increases the incidence of infections [2]. This threat is even more evident in institutionalized patients, indicating that factors other than age and immunosenescence, such as functional status and nutrition, are implicated in CAP morbidity [1,3].

Besides the high mortality after an episode of CAP, older adults also experience a decline in functional status (FS) [4,5,6]. The prevalence of functional decline ranges from 8.6% to 20% among CAP patients and is associated with prolonged hospital stays, episodes of early hospital readmission, and higher long-term mortality rates [4,6]. Moreover, FS and other factors, such as age, living alone, and cognitive impairment, have been shown to be strong predictors of institutionalization [7]. However, although hospitalization often precedes institutionalization, information on rates of nursing home placement after CAP admission and its specific risk factors is lacking [8].

Maintaining an adequate nutritional status is key to health and quality of life. However, older adults are susceptible to malnutrition due to physiological changes [9,10]. Malnutrition can be defined as an imbalance in nutrition that changes body composition and body cell mass, thereby decreasing physical and mental function and impairing clinical outcomes from disease [11]. The prevalence of malnutrition in older outpatients ranges from 1% to 24.6%, but is estimated to increase by 50% in rehabilitation settings, 20% in residential care, and 40% in hospitals [12].

Malnutrition in older adults is complex and has a multi-factorial origin [9]. One specific form of malnutrition especially difficult to screen and identify is a deficiency in micronutrients [9]. Micronutrient deficiencies in iron (Fe), vitamins C and D, vitamins B6 and B12, folate, and zinc have been linked to an impaired immune system [9]. As micronutrient status plays an important role in chronic disease and prevention, there is a need to quantify these deficiencies and identify subgroups at risk in the older population [10].

Therefore, although it seems that poor functional status and poor nutritional status are both the cause and consequence of CAP and the need for subsequent institutionalization, few studies have investigated functional decline in older patients after a hospital admission for CAP [6,13], and even fewer have addressed institutionalization and nutrition [3].

We hypothesized that functional decline and malnutrition are frequent problems in older patients after hospital admission for CAP, especially in those who will require institutionalization. We conducted the following study to assess the functional and nutritional status of older patients discharged from hospital for CAP, paying special attention to the main differences between institutionalized and community-dwelling patients, and the risk factors of three main mid-term adverse outcomes: functional decline, institutionalization, and malnutrition.

## 2. Methods and Patients

### 2.1. Study Design

A prospective cohort study was conducted at Hospital de la Santa Creu I Sant Pau, a tertiary university hospital in Barcelona, Spain. Patients admitted to hospital due to an episode of pneumonia were invited to participate. All participants gave written informed consent.

This study was approved by the hospital ethics committee. All study-related procedures were performed in accordance with the 1964 Helsinki declaration and its later amendments. This study is part of a prospective, observational, single-center project for which the aim is to evaluate the prognostic value of multiple biomarkers in older patients admitted for CAP (ClinicalTrials.gov; No.: NCT0462799). The project evaluates the prognostic value of immunophenotype in older patients who have been admitted for CAP and its association with other relevant clinical markers, especially those related to nutrition. Here, we analyzed the nutritional and functional status of participants 30–60 days after CAP diagnosis.

### 2.2. Subjects

Patients with radiologically confirmed pneumonia requiring hospitalization between May 2019 and July 2021 were invited to participate.

Pneumonia was defined as the presence of a new pulmonary infiltrate on X-ray or computed tomography and at least one of the following symptoms: fever, shivering, cough, expectoration, or malaise. The attending physician first evaluated the chest radiograph. At inclusion, the authors reviewed the radiological images to confirm the diagnosis. In previous studies, we showed a 95% diagnostic agreement with the radiologists [14], who were consulted in case of doubt. The attending physician determined all aspects of patient management. Exclusion criteria were hospitalization for 72 h or longer within the fifteen previous days, HIV virus infection, severe neutropenia (<1000/mL), chronic lymphocytic leukemia (because of their abnormal lymphogram), being a transplant recipient, and end-of-life clinical status.

### 2.3. Data Collection and Measures

At admission, we recorded demographic data, previous institutionalization, and the Pneumonia Severity Index (PSI) [15]. To assess functional status, we also asked about the Barthel index (BI) [16] in the 15 days prior to CAP to ensure that the premorbid functional status was not underestimated. Pathogens in samples obtained from sputum, blood, or other body fluids were studied using standard microbiological procedures. All patients included after March 2020 were tested for COVID-19 (SARS-CoV-2) using either the polymerase chain reaction or rapid antigenic test. In all patients included between January 2020 and March 2020, serologies were performed retrospectively and were negative in all cases.

Between 30 and 60 days after the diagnosis of pneumonia, a comprehensive geriatric assessment (CGA) and a general nutritional assessment (GNA) were performed at the Geriatric Day Hospital.

We recorded institutionalization rates (including both residential, long-term care, and intermediate-care centers over 30 days after admission), hospital readmission rates, Short Portable Mental Status Questionnaire (SPMSQ, ranges from 0—normal to 10—severe cognitive impairment) results [17], the Charlson Comorbidity Index (CCI) [18], the BI, and the number of instrumental activities of daily living (ADL) performed without assistance. Chronic kidney disease was assessed according to CCI (defined as a level of serum creatinine > 265 pmol/L, being on dialysis, being a receptor of renal transplantation, or presenting uremia), but all analyses were performed according to the definition of the KDIGO guidelines (glomerular filtration rate (GFR) of less than 60 mL/min per 1.73 m^2^) [19]. Functional decline was calculated as the difference between the current BI and the BI 15 days prior to CAP.

The GNA included body mass index (BMI), calculated as weight (kg) divided by the square of height (kg/m^2^), a blood test with nutritional parameters (albumin, vitamins of the B group, folate, zinc, vitamins C and D), and a malnutrition risk screening test using a revised version of the Mini Nutritional Assessment (MNA, Nestle Nutrition Institute) [20]. Nutritional status was considered adequate when MNA > 24; at risk for malnutrition when between 17 and 23.5; and protein–calorie malnutrition when < 17 [20]. CGA and GNA data, including blood tests, were obtained at the geriatric day hospital 30–60 days after CAP diagnosis.

Methods and techniques for micronutrient determinations and laboratory referent values are detailed in Supplementary File Text S1.

### 2.4. Statistical Analysis

Continuous variables are expressed as means and standard deviation (SD). Categorical variables are expressed as percentages relative to the total sample. Continuous variables were compared using the Mann–Whitney U test and categorical variables were compared with chi-square tests and Fisher’s exact test when necessary. Wilcoxon and McNemar tests were used to compare pairwise data. Variables with *p* values < 0.1 in univariate analyses that could be known at hospital discharge were selected for forward stepwise multivariate analysis to identify the predictors of functional decline, institutionalization, and malnutrition at 45 days of discharge. Malnutrition was defined as an MNA score < 17 points. Variables included in the final model were age, sex, previous BI, CCI, previous institutionalization, PSI without age, COVID-19 etiology, and length of stay.

We calculated sensitivity, specificity, and the positive and negative predictive value using an MNA score < 17 and/or presence of hypoalbuminemia. These indicators, which we assessed separately and together, were used to identify patients at greater risk of micronutrient deficiencies.

All calculations were performed using SPSS for Windows, version 27 (Armonk, NY, USA: IBM Corp), and *p* values of 0.05 were considered significant.

## 3. Results

**Figure 1:**
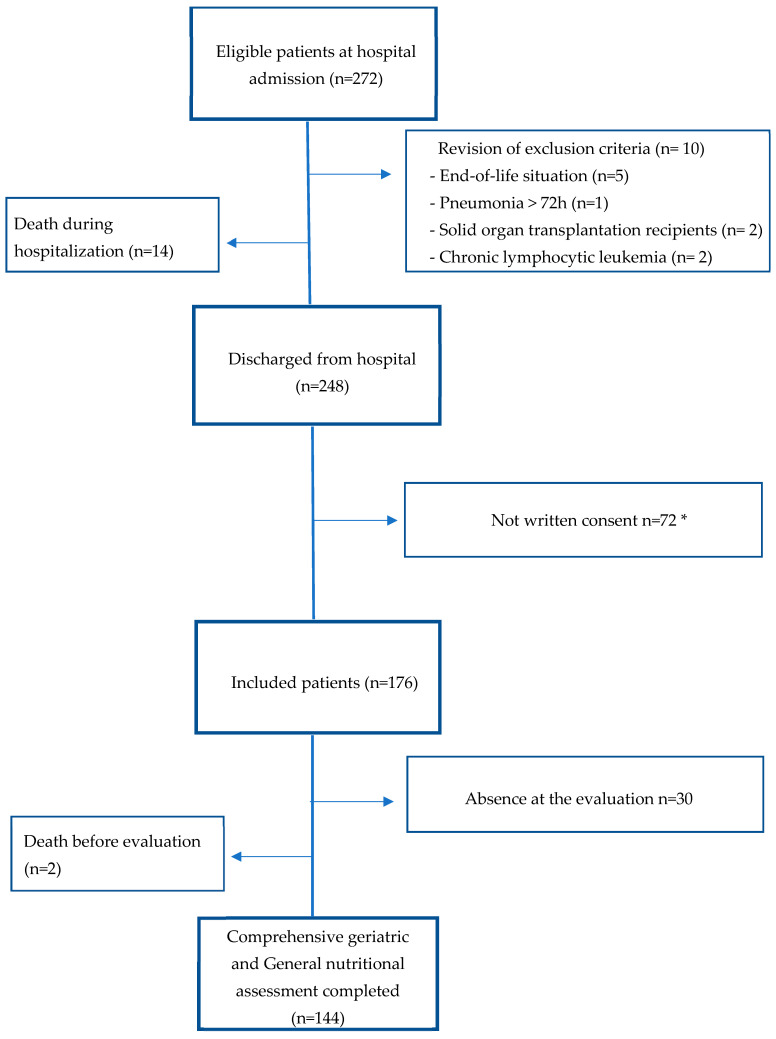
Study flowchart. * Most patients declined to consent or withdrew from the study in the context of the SARS-CoV-2 pandemic.

Two hundred and seventy-two patients were eligible for inclusion at hospital admission. Fourteen patients were excluded due to death during hospitalization, and ten were excluded when we revised the exclusion criteria a second time. Two hundred and forty-eight participants were invited to participate, but only one hundred and seventy-six patients accepted. Two patients died before evaluation, and thirty patients did not attend the evaluation. Most withdrawals from this study were due to lockdown in the context of the SARS-CoV-2 pandemic. A total of 144 patients completed the evaluation (see Figure 1).

Table 1 shows patient characteristics and differences between previously institutionalized and community-dwelling patients. Institutionalized patients prior to admission were older and showed a trend toward greater dependency for basic ADL, but the other characteristics recorded at admission were similar. The most common comorbidities were diabetes (mild to moderate and/or with chronic complications) (29.1%), chronic lung disease (28.4%), and cardiopathy (including congestive heart failure and acute myocardial infarction) (25.7%). The comorbidities are cited according to CCI.

Microbiological etiology was identified in 64 patients. The main etiologies were: SARS-CoV-2, 41 patients (28.5%); *Streptococcus pneumoniae,* 10 patients (6.8%); and *Legionella pneumoniae*, 7 patients (4.7%). Differences between patients with SARS-CoV-2 etiology and other etiologies are shown in Supplementary Table S1.

### 3.1. Functional Decline and Institutionalization after the CAP Episode

A comprehensive geriatric assessment was performed at 44.65 ± 14.4 days after discharge. The results are shown in Table 2. The CGA showed that patients who were institutionalized at 45 days after hospital discharge were more dependent for basic and instrumental ADL and had worse scores at SPMSQ than patients who returned to community dwelling. The GNA showed that although this group of patients was more frequently malnourished (67.9% vs. 9.5%), the risk of malnutrition was lower (28.6% vs. 45.7%) than that in patients who returned to community dwelling. Blood tests showed that presence of hypoalbuminemia was more prevalent in this group of patients (32.1% vs. 8.6%), and that albumin levels were lower, but without clinical significance (36.2 g/L vs. 39 g/L).

The BI was significantly lower than at baseline (83.75 ± 22.78 vs. 93.06 ± 17.13 *p* < 0.001). Seventy patients showed a functional decline of at least 5 points in BI (48.6%); a loss of over 10 points was found in forty-three patients (29.8%); and a loss of over 30 points was found in sixteen patients (11.1%). Table 3 shows patients’ characteristics regarding functional decline at hospital discharge.

In the multivariate analyses, age (OR 1.17, CI95% 1.09–1.26 *p* < 0.001), previous BI (OR 1.09, CI95% 1.05–1.14 *p* < 0.001), CCI (OR 1.56, CI95% 1.16–2.11, *p =* 0.003), length of stay (OR 1.1, CI 95% 1.02–1.18, *p =* 0.008), and previous institutionalization (OR 29.18, CI 95% 3.79–224.7 *p =* 0.001) remained predictors of functional decline of 10 points in BI. When the threshold of functional decline was defined as a difference of 5 points or 30 points in BI (instead of 10 points), predictors of functional decline did not change.

Twenty-eight patients were institutionalized (19.4%) vs. thirteen (9%) prior to CAP admission, *p* < 0.001. One patient institutionalized prior to admission returned to community dwelling. Among the 131 previously community-dwelling patients, 16 (12.1%) were institutionalized after CAP. The main differences between these patients and patients returning to community dwelling are shown in Table 3. In the multivariable analyses, previous BI (OR 0.96, CI 95% 0.93–0.99 *p =* 0.014) and length of stay (OR 1.06, CI 95% 1.00–1.13 *p =* 0.041) were independently associated with new institutionalization.

### 3.2. General Nutritional Assessment

The nutritional assessment is shown in Table 2. Multivariate analyses showed that malnutrition (MNA score < 17 points) could be predicted at discharge from previous BI (OR 0.95, CI95% 0.92–0.98 *p =* 0.005), previous institutionalization (OR 10.62, CI95% 2.20–51.21 *p =* 0.003), and length of stay (OR 1.12, CI95% 1.045–1.20 *p =* 0.002). Micronutrient deficiency was highly prevalent, as shown in Table 4. The most common deficiencies were zinc (61.8%) followed by vitamin D (54.1%) and vitamin C (45.1%).

To identify patients with a greater risk of micronutrient deficiency, we distributed patients into three groups: MNA < 17 points (30 patients, 20.8%) vs. MNA >17 points (114 patients, 79.1%); hypoalbuminemia (19 patients, 13.2%) vs. albumin >= 35 g/L (125 patients, 86.8%); and malnourished patients (either MNA < 17 or presenting hypoalbuminemia; 39 patients, 27%) vs. well-nourished (MNA > 17 and albumin > 35 g/L; 105 patients, 72.9%).

In the group with an MNA < 17, the main deficiencies were zinc in 26 patients (86.6%), folate in 7 (23.3%), vitamin C in 19 (63.3%), and vitamin D in 25 (83.3%). In the hypoalbuminemia group, the main deficiencies were 16 (84.2%), 4 (21%), 14 (73.6%), and 78 (54.1%), respectively. An MNA < 17 points and hypoalbuminemia showed good specificity to detect zinc, folic acid, vitamin C, and vitamin D deficiencies (Supplementary Tables S2 and S3). Thirty-nine patients (27%) presented either hypoalbuminemia or an MNA score < 17 points. These two indicators were used to identify patients at greater risk of presenting micronutrient deficiencies (Table 5).

## 4. Discussion

This prospective study shows that 45 days after admission for CAP, half of the patients had functional decline and one in five were institutionalized. The main risk factors were age, previous institutionalization and functional status, comorbidity, and length of hospital stay. Even more striking was the high rate of malnutrition, which affected one in five participants and over half of the institutionalized patients. Micronutrient deficiencies deserve special attention, with vitamin D, vitamin C, and zinc being the most prevalent. These micronutrient deficiencies were more frequent in patients with either an MNA score suggestive of undernutrition or hypoalbuminemia. This is of note, as these analyses are not routinely performed in most laboratories. Therefore, our findings provide valuable information on the evolution of older adults after admission for CAP that could help us improve their follow-up and long-term prognosis.

CAP has an interesting relationship with functional status in older adults, both because of its key role as a predictor of short- and long-term outcomes and because it is a common cause of functional decline in this population [6]. In our study, half of the patients experienced a functional decrease of at least 5 Barthel Index (BI) points, a third showed a clinically relevant decrease of at least 10 points, and one in ten had a catastrophic decrease of 30 or more points. The functional decline in our patients was higher than the one in five at 30 days previously reported in similar pre-COVID-19 studies [21,22], but it is in line with the results of the study by Le Gentil et al., who reported a 3-month persistent functional decline of 42% in older adults admitted for pneumonia in 2020 [23]. Moreover, consistent with these observations and those of other authors, we did not find that COVID-19 etiology was a statistically significant independent factor for functional decline, while age [23], previous institutionalization and a better score in BI [24], Charlson Index (CCI) [24], and length of stay [23,24] were risk factors. We consider that restrictions due to pandemic lockdowns—such as isolation, limited mobility, and little physical therapy—contributed to greater functional decline even in patients with a higher BI. It is likely for this reason and for the “floor effect” that a better BI was a risk factor for functional decline in recent studies.

As in previous studies, 12% of our patients were institutionalized at 45 days [8]. Although CAP is an acute and treatable disease, the burden of continued institutionalization after a CAP admission is lower than that for hip fracture or stroke but higher than that for myocardial infarction or other hospitalizations [25]. However, to the best of our knowledge, there is little literature that specifically analyzes risk factors after CAP admission. We found well-known, non-modifiable risk factors for institutionalization after general hospital admission, such as age and a lower BI, but also length of stay, which is a potentially modifiable factor.

Most patients in our study were in their late seventies. They were autonomous and had low comorbidity. But, our study is of special interest in that it provides detailed characteristics of patients who were admitted from and discharged to institutions. Institutionalized patients prior to admission were older and showed a trend toward greater dependency for basic ADL, but the other characteristics recorded at admission were similar. Patients who were newly institutionalized after admission had greater dependency and more chronic renal impairment. No other differences were observed. However, the CGA at 45 days of CAP admission gave us much more information. Institutionalized patients were not only older and more dependent for basic and instrumental ADL, but they also had more cognitive impairment, more inflammation, and higher malnourishment. As we do not have a geriatric or nutritional assessment on admission, we cannot establish any causal relationship, but functional and cognitive decline and especially malnutrition are potentially modifiable factors that deserve attention.

The most striking results of our study are those related to nutritional assessment 45 days after CAP. Although most of the patients were overweight, according to the MNA, 21% were malnourished and 42% were at high risk of malnutrition. These numbers rose to 67.9% and 28.6%, respectively, in institutionalized patients. In the previous literature, the reported rates of malnutrition and risk of malnutrition are, respectively, 7.9% and 28.9% [26] in the community, 18.5% and 49% [27] in nursing homes, and 17.2% and 48.5% [28] in hospitals. Our malnutrition rates after a hospital stay for CAP are thus clearly higher than expected. A mean post-hospital discharge MNA of 22 points is similar to that described in previous studies [28,29].

Nutrition has been well-studied in in-hospital patients with CAP for its relationship with frailty [30] and dysphagia [31] and as a risk factor for poor outcomes [32]. Nevertheless, little is known about nutritional status in older adults after CAP discharge except in relation to COVID-19 pneumonia. Reports of COVID-19 patients at hospital discharge have shown malnutrition in 18% and risk of malnutrition in 62%, with bedridden patients having even worse nutritional status [33]. In our study, the risk factors for malnutrition were previous dependency for basic ADL, institutionalization, and, again, an important, potentially modifiable risk factor, length of hospital stay. COVID-19 was not a statistical risk factor for malnutrition.

We detected significant deficiencies in micronutrients. The most frequent were vitamin D, zinc, and vitamin C. These three micronutrients play roles in antioxidant, anti-inflammatory, antithrombotic, antiviral, and immuno-modulatory functions and are useful in both innate and adaptive immunity. For these reasons, their protective and therapeutic functions against COVID-19 infection have been explored in recent articles, which found that they can be involved in relevant outcomes, such as mortality, respiratory distress, disease severity, and prolonged hospital stay [34]. Their role in CAP has been studied previously [35,36,37], but we do not have data concerning these micronutrient deficiencies in older adults after a CAP admission. Deficiencies reported for the general older population are 43% for vitamin D (<50 nmol/L) [38], 12% for vitamin C, and 10.8% for zinc [39]. Thus, deficiencies observed in our patients clearly exceed those expected. These deficiencies could be relevant, especially considering that adequate supplementation with the usual diet is difficult in this population. An additional problem in clinical practice is that these serum micronutrients are not routinely analyzed in most laboratories. Although further research is needed, our findings suggest that supplementation could be considered in patients with an MNA < 17 or hypoalbuminemia. Nevertheless, MNA < 17 or hypoalbuminemia show low sensitivity, and deficiencies in other patients may remain undetected.

Our study has several limitations. First, the study population was limited to a single community hospital. Second, it was difficult to complete follow-up in all recruited patients. However, because it is not always easy for older patients to attend hospital visits, we established a period of 30–60 days to ensure clinical stability and to facilitate their attendance. Third, the emergence of the COVID-19 pandemic during the inclusion period was a major challenge to recruitment. In the midst of the emergence of COVID-19, we were faced with the dilemma of how to proceed with the study of a new respiratory pathogen that would likely change the epidemiological scenario during the study period. The inclusion/exclusion criteria of this study did not contemplate the etiology of CAP. Therefore, we decided to include patients with COVID-19 CAP who met the inclusion criteria while studying and accurately documenting the etiology of SARS-CoV-2 in all patients. Because of possible concerns about the influence of this patient heterogeneity on the results, COVID-19 etiology was entered as a variable in all the multivariate analyses but did not reach statistical significance.

The main strengths of this study are its prospective design, the detailed comprehensive geriatric and nutritional assessments, and the evaluation of functional decline and institutionalization.

## 5. Conclusions

Our study reflects the profile of older adults after hospitalization for CAP by describing their main mid-term difficulties: functional decline, institutionalization, and malnutrition. A longer hospital stay was a risk factor for these three poor outcomes, and patients with previous dependency for basic ADL were more susceptible to malnutrition and institutionalization. Our results emphasize the importance of adhering to quality standards and geriatric interventions to reduce hospital stay. Although the length of hospital stay could be a surrogate marker of other geriatric characteristics not collected at the time of admission, this readily available data can identify those patients who require further evaluation. We suggest a nutritional assessment should be conducted in the follow-up of older patients after CAP and micronutrients should be considered, especially in patients who present hypoalbuminemia or an MNA score < 17.

## Figures and Tables

**Table 1:** Patients’ main baseline characteristics, and differences between previously institutionalized and community-dwelling patients.

Baseline Characteristics	*N* = 144	Previous Institutionalization *N* = 13	Previous Community Dwelling *N* = 131	*p*
Male sex, *n* (%)	80 (55.6)	5 (38.4)	75 (57.2)	0.193
Age, mean years (±SD)	77.1 (7.9)	81.5 (8.6)	76.7 (7.7)	**0.046**
Smoking status, *n* (%)	7 (4.9)	0	7 (5)	0.392
Barthel Index, mean points (±SD)	93.0 (17.1)	78.0 (31.4)	94.5 (14.3)	0.064
SARS-CoV-2 etiology, *n* (%)	41 (28.4)	1 (7.6)	40 (30.5)	0.082
PSI, mean points (±SD)	98.1 (25.9)	111.2 (25.0)	96.8 (25.7)	**0.025**
PSI without age, mean points (±SD)	20.9 (23.5)	29.6 (28.3)	20.1 (22.9)	0.166
Intensive care admission, *n* (%)	12 (8.3)	1(7,6)	11(8,3)	0.930
Length of stay, mean days (±SD)	9.7 (7.8)	9.9 (7.3)	9.6 (7.9)	0.810
Charlson Comorbidity Index, mean points (±SD)	1.5 (1.6)	1.0 (1.8)	1.0 (1.6)	0.751
**Main comorbidities** ^a^**:**				
Chronic lung disease, *n* (%)	42 (28.4)	3 (23)	38 (29)	0.651
Mild to moderate diabetes ^b^, *n* (%)	33 (22.3)	1 (7.6)	32 (24.4)	0.171
Congestive heart failure, *n* (%)	24 (16.2)	3 (23)	20 (15.2)	0.463
Acute myocardial infarction, *n* (%)	14 (9.5)	1 (0.07)	12 (9.1)	0.896
Cerebrovascular disease, *n* (%)	14 (9.5)	0	14 (10.6)	0.215
Peripheral vascular disease, *n* (%)	10 (6.8)	2 (15.3)	8 (6.1)	0.209
Diabetes with chronic complications ^c^, *n* (%)	10 (6.8)	1 (7.6)	9 (6.8)	0.911
Malignant tumors, *n* (%)	9 (6.1)	1 (7.6)	8 (6.1)	0.822
Dementia, *n* (%)	8 (5.4)	2 (15.3)	6 (4.5)	0.105
Lymphoma, *n* (%)	5 (3.4)	0	5 (3.8)	0.473
Rheumatic disease, *n* (%)	4 (2.7)	1 (7.6)	3 (2.2)	0.258
Peptic ulcer, *n* (%)	3 (2)	0	3 (2.2)	0.581
Solid metastatic tumor, *n* (%)	2 (1.4)	0	2 (1.5)	0.654
Mild liver disease, *n* (%)	2 (1.4)	0	2 (1.5)	0.654
Chronic kidney disease ^d^, *n* (%) Polycythemia Vera	2 (1.4) 2 (1.4)	1 (7.6) 0	1 (0.7) 2 (1.5)	**0.042** 0.654

PSI: Pneumonia Severity Index; *p* values < 0.005 are marked in bold ^a^ According to Charlson Comorbidity Index; ^b^ Mild to moderate diabetes, defined as patients requiring insulin or oral antidiabetics; ^c^ Diabetes with chronic complications, defined as patients with complications, such as retinopathy, neuropathy, or nephropathy, attributable to diabetes; ^d^ Chronic kidney disease according to Charlson Comorbidity Index, defined as level of serum creatinine > 265 pmol/L, being on dialysis, being a receptor of renal transplantation, or presenting uremia.

**Table 2:** Comprehensive geriatric assessment and general nutritional assessment, and main differences between institutionalized patients and community-dwelling patients at an average of 45 days post-discharge.

Comprehensive Geriatric Assessment	*N* = 144	Institutionalization at 45 d after Discharge *N* = 28	Community Dwelling at 45 d after Discharge *N* = 116	*p*
Barthel Index, mean points (±SD)	83.7 (22.7)	54.0 (25.7)	90.5 (14.7)	**<0.001**
Independence for instrumental activities of daily living, mean points (±SD)	4.4 (2.6)	1.07 (1.1)	5.11 (2.2)	**<0.001**
Hospital readmission after episode of pneumonia, *n* (%)	10 (6.9)	2 (7.1)	8 (6.8)	0.963
SPMSQ, mean points (±SD)	1.2 (1.9)	2.4 (6.3)	1.0 (1.7)	**<0.001**
**General nutritional assessment at 45 days of discharge:**				
BMI, kg/m^2^, mean points (±SD) ^a^	27.0 (5.4)	25.0 (6.3)	27.3 (5.2)	**0.016**
MNA questionnaire, mean points (±SD):	20.51 (5.0)	14.7 (4.7)	21.6 (4.2)	**<0.001**
Well-nourished (>24 points), *n* (%)	53 (37)	1 (3.6)	52 (44.8)	**<0.001**
Risk of malnutrition (17–24), *n* (%)	61 (42)	8 (28.6)	53 (45.7)	**<0.001**
Malnourished (<17 points), *n* (%)	30 (21)	19 (67.9)	11 (9.5)	**<0.001**
Hypoalbuminemia (<34.9 g/L), *n* (%)	19 (13.2)	9 (32.1)	10 (8.6)	**<0.001**
Lymphopenia (<1000 × 109/L), *n* (%)	23 (16)	4 (14.3)	19 (16.4)	0.786
Chronic kidney disease ^b^	119 (82.6)	23 (82.1)	96 (82.7)	0.938
eGFR level 60–90 mL/min, *n* (%)	82 (56.9)	12 (42.8)	70 (60.3)	**0.037**
eGFR level 30–60 mL/min, *n* (%)	28 (19.4)	6 (21.4)	22 (18.9)	
eGFR level 15–30 mL/min, *n* (%)	8 (5.6)	4 (14.2)	4 (3.4)	
eGFR level <15 mL/min, *n* (%)	1 (0.7)	1 (3.5)	0	
Albumin (g/L), mean points (±SD)	38.8 (3.9)	36.2 (4.2)	39.0 (3.6)	**<0.001**
CRP (mg/L), mean points (±SD)	11.5 (22.5)	24.54 (33.9)	9.67 (19.2)	**<0.001**
Sodium (mmol/L), mean points (±SD)	139.8 (3.9)	139.6 (3.9)	139.9 (2.6)	0.466
Creatinine (pmol/L), mean points (±SD)	90.8 (43.6)	106.3 (70.0)	87.0 (33.9)	0.824
Hemoglobin (g/dL), mean points (±SD)	124.5 (18.0)	120.1 (16.9)	124.92 (18.7)	0.149

*p* values < 0.005 are marked in bold. SPMSQ, Short Portable Mental Status Questionnaire; CRP, C-reactive protein; ^a^ BMI, body mass index, calculated as weight (kg) divided by the square of height ^2^ (m^2^); ^b^ Chronic kidney disease according to KDIGO guidelines.

**Table 3:** Main differences between patients with functional decline > 10 points and patients with no functional decline and between new institutionalization and return to community dwelling within 45 days after discharge from an episode of CAP.

	Functional Decline (>10 Points) *N* = 43	No Functional Decline *N* = 101	*p*	New Institutionalization *N* = 16	Return to Community Dwelling *N* = 115	*p*
Male sex, *n* (%)	9 (56.2)	66 (57.3)	0.931	9 (55.6)	66 (57.3)	0.931
Age, mean points (±SD)	82.1 (7.5)	75.0 (7.1)	**0.001**	80.0 (7.4)	76.2 (7.6)	0.063
Smoking status, n (%)	2 (4.6)	5 (4.9)	0.939	2 (12.5)	5 (4.3)	0.174
Hospital admission after episode of CAP, *n* (%)	6 (13.9)	11 (10.8)	0.602	2 (12.5)	8 (6.8)	0.434
Previous Barthel Index, mean points (±SD)	96.4 (15.5)	91.6 (17.6)	**0.005**	82.5 (25.6)	96.2 (11.2)	**0.031**
SARS-CoV-2 etiology, n (%)	5 (11.6)	36 (35.6)	**0.003**	0	40 (34.7)	**0.005**
PSI, mean points (±SD)	113.4 (26.2)	91.6 (23.0)	**<0.001**	103.94 (24.0)	95.8 (25.9)	0.112
PSI without age, mean points (±SD)	31.2 (26.5)	16.5 (20.7)	**<0.001**	23.9 (21.8)	19.5 (23.1)	0.329
Intensive care admission, *n* (%)	5(11.6)	7(6.9)	0.351	3 (18.7)	8 (6.9)	0.111
Length of stay, mean days (±SD)	12.7 (8.5)	8.4 (7.2)	**<0.001**	15.1 (9.0)	8.9 (7.5)	**0.001**
Charlson Comorbidity Index, mean points (±SD)	2.1 (1.8)	1.2 (1.4)	**<0.001**	2.1 (2.2)	1.4 (1.5)	0.372
**Main comorbidities:**						
Chronic lung disease, *n* (%)	13 (30.2)	28 (27.7)	0.760	4 (25)	34 (29.5)	0.706
Diabetes (mild to severe), *n* (%)	15 (34.8)	27(26.7)	0.325	6 (37.5)	34 (29.5)	0.518
Cardiopathy ^a^, *n* (%)	19 (44.1)	11 (10.8)	**<0.001**	4 (25)	22 (19.1)	0.518
Chronic kidney disease ^b^, *n* (%)	1 (2.3)	1 (0.9)	0.531	1 (6.2)	0	**0.007**
Cerebrovascular disease, *n* (%)	6 (13.9)	8 (7.9)	0.263	2 (12.5)	12 (10.4)	0.802
Peripheral vascular disease, *n* (%)	7 (16.2)	3 (2.9)	**0.004**	3 (18.7)	5 (4.3)	**0.024**
Malignant tumors or solid metastatic tumors, *n* (%)	4 (9.3)	7 (6.9)	0.624	2 (12.5)	8 (6.9)	0.434
Dementia, *n* (%)	4 (9.3)	4 (3.9)	0.200	1 (6.2)	5 (6.2)	0.733

PSI: Pneumonia Severity Index, CAP: community-acquired pneumonia; *p* values < 0.005 are marked in bold ^a^ Cardiopathy includes patients with myocardial infarction and congestive heart failure; ^b^ Chronic kidney disease according to the Charlson Comorbidity Index, defined as a level of serum creatinine > 265 pmol/L, being in a dialysis program, being a receptor of renal transplantation, or presenting uremia.

**Table 4:** Vitamin levels.

Vitamins				
	Mean Value (±SD)	Deficiency Definitions	Deficiency *n*, (%)	Normal *n*, (%)
Vitamin B1 (nmol/L)	137 (41.1)	<66.5 nmol/L	3/141 (2.1)	138/141 (97.8)
Vitamin B2 (ng/mL)	187.8 (38.6)	<125 ng/mL	4/139 (2.8)	135/139 (93.8)
Vitamin B6 (ng/mL)	153.6 (544.7)	<125 ng/mL	2/141 (1.4)	139/141 (98.5)
Folate (nmol/L)	16.8 (9.6)	<7 nmol/L	11/144 (7.6)	133/144 (92.3)
Vitamin B12 (pmol/L)	343.1 (265.5)	<150 pmol/L	5/143 (3.5)	138/143 (96.5)
Vitamin C (mg/dL)	0.55 (0.45)	<0.4 mg/dL	65/136 (45.1)	73/136 (54.0)
Vitamin D (nmol/L) *	50.2 (30.9)	<50 nmol/L	78/144 (54.1)	26/144 (18.1)
Zinc (pmol/L)	10.1 (2.3)	<8.4 pmol/L	89/144 (61.8)	55/144 (38.2)

Micronutrient deficiencies were defined according to the hospital laboratory reference values (see Supplementary File Text S1). * Vitamin D insufficiency was defined as levels >50 nmol/L but <75 nmol/L.

**Table 5:** Sensitivity, specificity, positive predictive value, and negative predictive value of detecting micronutrient deficiencies in MNA < 17 or hypoalbuminemia.

Hypoalbuminemia or MNA < 17 *N* = 39/144	Zinc	Folate	Vitamin C	Vitamin D *
Sensitivity (%)	35.9	72.7	40.0	28.8
Specificity (%)	87.2	76.6	84.9	87.1
Positive predictive value (%)	82.0	20.5	70.2	87.2
Negative predictive value (%)	45.7	97.1	61.3	20.0

* Vitamin D insufficiency defined as levels < 75 nmol/L.

## Data Availability

The datasets used and/or analyzed during the current study are available from the corresponding author upon reasonable request.

## Supplementary Materials

The following are available online at https://www.mdpi.com/article/10.3390/nu16010011/s1, Text S1. referent values; Table S1. Main differences between COVID and non-COVID patients; Table S2. Sensitivity, Specificity, Positive Predictive value and Negative Predictive Value to detect main micronutrient deficiencies in MNA score < 17 points group; Table S3. Sensitivity, Specificity, Positive Predictive value and Negative Predictive Value to detect main micronutrient deficiencies in Hypoalbuminemia group.

## Abbreviations

CAP	Community-acquired pneumonia
FS	Functional status
BI	Barthel Index
PSI	Pneumonia Severity Index
CGA	Comprehensive geriatric assessment
GNA	General nutritional assessment
SPMSQ	Short Portable Mental Status Questionnaire
CCI	Charlson Comorbidity Index
ADL	Activities of daily living
BMI	Body mass index
MNA	Mini Nutritional Assessment
