# Supplementary material for: Malnutrition, Functional Decline, and Institutionalization in Older Adults after Hospital Discharge Following Community-Acquired Pneumonia
Source: Nutrients. 2023 Dec 20;16(1):11. doi: 10.3390/nu16010011 (PMC10780721; doi:10.3390/nu16010011)
Supplement: Supplementary file 1 [file nutrients-16-00011-s001.zip › nutrients-2758431-supplementary.pdf]

Text S1: referent values

Vitamin B1 and vitamin B6 were measured using high performance liquid chromatoography (HPLC) coupled to a fluorescence detector (Waters, Manchester, UK). Vitamin B2 was measured with the LC-MS/MS system, using an "Agilent HPLC coupled to Triple Quadrupole (TQD) mass spectrometer (Agilent Technologies, Santa Clara, USA). Vitamin C was measured using an ultraperformance liquid chromatography (UPLC) system coupled to a Waters Resolve C18 (Waters, Manchester, UK) with electrochemical detection. Vitamin B12 and folate were measured with an Alinity ci-series (Abbott Laboratories, Chicago, IL, USA) using the Chemiliuminescent Microparticle Immuno Assay (CMIA). Zinc was measured by flame atomic absorption spectroscopy with Agilent AA Duo Atomic Absorption System (Agilent Technologies, Santa Clara, USA). Serum 25-hydroxyvitamin D (vitamin D) concentration was measured by the LC-MS/MS system, using a Waters ACQUITY 1 ultra performance liquid chromatograph (UPLC) coupled to a Waters Xevo TQD mass spectrometer (Waters, Manchester, UK).

Micronutrient deficiencies were defined according to the hospital laboratory reference values: vitamin B1 < 66.5 nmol/l, vitamin B2 < 125 ng/ml, vitamin B6 125 ng/ml, vitamin B12 150 pmol/l, folic acid < 7 nmol/l, vitamin C <0.4 mg/dl and vitamin D < 50 nmol/l (vitamin D insufficiency < 75 nmol/l). Hypoalbuminemia was defined as levels of plasma albumin < 34.9g/l.

Table S1: Main differences between COVID and non-COVID patients

**Baseline characteristics**	**N = 144**	**COVID N= 41**	**No COVID N= 103**	**p**
Male sex, n (%)	80 (55.6)	23 (56.0)	57 (55.3)	0.934
Age, mean (±SD)	77.1 (7.9)	73.3 (5.2)	78.6 (8.3)	**<0.001**
Previous Institutionalization, n (%)	13 (9)	1 (2)	12 (11.6)	0.08
Smoking status, n (%)	7 (4.9)	0	7 (6.7)	0.087
Previous hospital admission, n (%)	17 (9.7)	2 (4.8)	15 (14.5)	0.104
Barthel index, mean points (±SD)	83.7 (22.7)	96.7 (10.4)	91.6(19.0)	0.112
PSI, mean points (±SD)	98.1 (25.9)	84.5 (17.4)	103.5 (26.8)	**<0.001**
PSI without age, mean points (±SD)	20.9 (23.5)	11.2 (15.3)	24.8 (25.1)	**<0.001**
Intensive care admission n (%)	12 (8.3)	2 (5.1)	10 (9.7)	0.344
Length of stay, mean points (±SD)	15.1 (9.0)	8.0(7.3)	10.3 (8.0)	**0.045**
Charlson comorbidity index mean points (±SD)	1.5 (1.6)	0.85 (1.0)	1.79 (1.7)	**0.002**

Table S2. Sensitivity, Specificity, Positive Predictive value and Negative Predictive Value to detect main micronutrient deficiencies in MNA score < 17 points group

**MNA<17**<br>**N=30**	**Zinc**	**Folate**	**Vitamin C**	**Vitamin D***
Sensitivity (%)	29.2	63.3	29.3	21.1
Specificity (%)	92.7	82.7	87.6	80.7
Positive predictive value (%)	86.6	23.3	67.8	83.3
Negative predictive value (%)	44.7	96.4	58.1	18.4

*Vitamin D insufficiency defined as levels < 75 nmol/l

Table S3. Sensitivity, Specificity, Positive Predictive value and Negative Predictive Value to detect main micronutrient deficiencies in Hypoalbuminemia group

**Hypoalbuminemia**<br>**N=19**	**Zinc**	**Folate**	**Vitamin C**	**Vitamin D***
Sensitivity (%)	17.9	36.3	21.5	15.1
Specificity (%)	94.5	88.7	93.1	96.1
Positive predictive value (%)	84.2	21	73.6	94.7
Negative predictive value (%)	41.6	94.4	57.1	21

*Vitamin D insufficiency defined as levels < 75 nmol/l
